# Contributions of androgen and estrogen to fetal programming of ovarian dysfunction

**DOI:** 10.1186/1477-7827-4-17

**Published:** 2006-04-10

**Authors:** David H Abbott, Vasantha Padmanabhan, Daniel A Dumesic

**Affiliations:** 1 National Primate Research Center, Department of Ob/Gyn, and Endocrinology-Reproductive Physiology Training Program, University of Wisconsin, Madison, WI 53715, USA; 2 Departments of Pediatrics, Ob/Gyn, Cellular and Integrative Physiology, and Reproductive Sciences Program, University of Michigan, Ann Arbor, MI 48109, USA; 3 Reproductive Medicine & Infertility Associates, Woodbury, MN 55125, USA

## Abstract

In female mammals, including humans, deviations from normal androgenic or estrogenic exposure during fetal development are detrimental to subsequent adult ovarian function. Androgen deficiency, without accompanying estrogen deficit, has little apparent impact on ovarian development. Fetal estrogen deficiency, on the other hand, results in impaired oocyte and follicle development, immature and abnormal adult ovaries, and excessive ovarian stimulation from endogenous gonadotropins ultimately generating hemorrhagic follicles. Complete estrogen deficiency lasting into adulthood results in partial ovarian masculinization. Fetal androgen excess, on the other hand, mediated either by direct androgen action or following androgen aromatization to estrogen, reprograms ovarian development and reproductive neuroendocrinology to mimic that found in women with polycystic ovary syndrome: enlarged, polyfollicular, hyperandrogenic, anovulatory ovaries with accompanying LH hypersecretion. Oocyte developmental competence is also compromised. Insulin is implicated in the mechanism of both anovulation and deficient oocyte development. Fetal estrogen excess induces somewhat similar disruption of adult ovarian function to fetal androgen excess. Understanding the quality of the fetal female sex steroid hormone environment is thus becoming increasingly important in improving our knowledge of mechanisms underlying a variety of female reproductive pathologies.

## Introduction

Impaired fetal growth and subsequent adult cardiovascular disease [1] provide the best known example of fetal programming of adult pathology. Fetal programming is triggered when a stimulus or insult occurs at a gestational age critical for target organ differentiation, growth or development and induces permanent changes in organ size, structure or function. Developmental insults to the fetus, such as low calorie or low protein maternal diets, program for hypertension, insulin resistance, type 2 diabetes and obesity in adulthood [2]. Fetal glucocorticoid excess also programs many of the same adult traits induced by low calorie and low protein maternal diets, and has been proposed as the common physiological mechanism translating maternal environmental factors into impaired fetal growth and fetal programming of organ function [3].

Steroid hormone excess, including glucocorticoid and sex hormone excess during fetal life, is well known to induce permanent alterations in adult female phenotype. For example, fetal androgen excess induces female uro-genital virilization along with masculinized behavior, and neural anatomy and function [4], while fetal estrogen excess results in female reproductive tract abnormalities, including clear cell adenocarcinoma of the vagina and cervix, as well as increased risk of breast cancer [5]. Less is known, however, about fetal progestin excess which can mimic androgen excess in its fetal programming outcomes [6,7]. Steroid hormones mediate their classical or genomic actions by binding to nuclear receptors and engaging a variety of molecular chaperones permitting ultimate binding of the entire complex to steroid response elements on promoters of target genes [8]. Nonclassical or non-genomic actions of steroids, including rapid changes in cytoplasmic protein function without steroid-receptor complex binding to DNA, are mediated by cell membrane receptors such as GPR30, a G-protein coupled protein mediating rapid estrogen action (estrogen membrane receptor: [9]; androgen membrane receptor: [10]; progesterone membrane receptor: [11]). Whether prenatal steroid hormone programming of postnatal ovarian function involves either or both classical and non-classical action is not known. Studies to date have assumed a classical or genomic action.

It is certainly becoming clear that exposing female fetuses to androgen excess results in enlarged adult ovaries that are polyfollicular, anovulatory and hyperandrogenic, and resemble those found in women with polycystic ovary syndrome (PCOS: monkeys: [12]; sheep: [13]). Fetal estrogen excess, on the other hand, diminishes ovarian size and function while increasing anovulation in adulthood [14-16]. This mini-review will focus on the role of fetal androgens and estrogens in determining postnatal ovarian phenotype (summarized in Table 1), and the relevance of such fetal programming to reproductive health in women.

**Table 1:** Summary of major reproductive dysfunction or anomalies associated with fetal deficiency or excess of androgen or estrogen

**Major reproductive dysfunction/anomaly**	**Fetal deficiency of:**		**Fetal excess of:**	
	**Androgen^b^**	**Estrogen^a^**	**Androgen**	**Estrogen**
**Ovarian**				
Reduced ovulatory frequency	+ [29]	+ [45]	+ [12, 50–56]	+ [15, 76, 80, 81]
Reduced follicle number	?	+ [33, 44]	+ [69]	+ [14, 77]
Reduced ovarian response to FSH	+ [29]	+ [42, 43]	+ [69]	?
Excessive, endogeneous hypergonadotropic-inducing hemorrhagic follicles	-	+ [38, 45]	-	-
Ovarian hyperandrogenism	-	+ [38]	+ [49, 53]	+ [77, 79]
Polycystic ovaries	?	-	+ [13, 52, 57]	?
Impaired oocyte developmental competence	?	?	+ [28, 69, 70]	?
Partial masculinization of the ovary	-	+ [47, 48]	-	-
Premature ovarian senescence	?	?	+ [50–52]	?
**Neuroendocrine**				
Increased LH levels	?	+ [24, 38, 45]	+ [12, 53, 56, 60]	?
Increased FSH levels	?	+ [24, 45]	-	?
De-sensitized estradiol/progesterone negative feedback on LH	?	?	+ [49, 53–56, 58–60]	?
Increased gonadotrope LH sensitivity to GnRH	?	?	+ [49]	?

a : as discussed in the text, many instances of fetal androgen and estrogen deficiencies persist into adult life and partially confound assessments of the precise causes of the reproductive abnormalities found.

b : abnormalities summarized in this table reflect studies in which there were no concomitant deficiencies in estrogen

## Fetal androgen deficiency

Within the ovary, androgens are synthesized mainly within theca cells and the ovarian stroma, while mural granulosa cells convert theca-cell derived androstenedione into testosterone and dihydrotestosterone (DHT). Since androgen receptors are expressed in the fetal ovary [17] and in oocytes, granulosa and theca cells, and ovarian stroma in the mature, adult ovary [18-20], androgen action can effect many components of ovarian development and cyclical function [21].

Discrete experimental induction of fetal androgen deficiency induced by the administration of flutamide to pregnant rhesus monkeys during either early or late gestation fails to alter age at menarche and at first ovulation in exposed female fetuses [22]. Fetal deficiencies in female androgen biosynthesis induced by congenital defect or experimentally induced knockout of mitochondrial steroidogenic acute regulatory protein (StAR; [23]), and steroid biosynthetic enzyme, P450c17 [24,25], however, produce obvious defects in ovarian function, but androgen deficiencies also persist into postnatal life. Androgen deficiencies in adult female StAR knockout mice result in undectectable circulating levels of testosterone, extremely low levels of progesterone and corticosterone, and a poorly developed reproductive tract, indicative of hypoestrogenism [23]. Not surprisingly, the ovaries fail to show major follicle development or the presence of corpora lutea, but they do become enlarged due to hypertrophy of the stroma under endogenous hypergonadotropic over-stimulation. Similar to the androgen deficiency produced by defective or absent StAR, women deficient in the biosynthetic enzyme, P450c17, show marked impairment in androgenic, estrogenic and glucocorticoid biosynthesis [26], as well as anovulatory, hypergonadotropic hypogonadism. The ovaries are devoid of major follicle development, but experimentally-induced gonadotropic ovarian hyperstimulation for *in vitro *fertilization (IVF) induces the growth of dominant follicles that yield fertilizable oocytes at retrieval. None of the fertilized oocytes, however, progress beyond the 7-cell embryonic stage [24], and fail to reach developmental stages when they are completely dependent on the embryonic genome [27]. Such impaired embryonic developmental competence probably reflects a sub-optimal intra-follicular estrogenic environment due to deficient production of androgen precursors for estrogen biosynthesis by mural granulosa cells [28].

When life-long androgen deficiencies do not concomitantly result in fetal estrogen deficiencies, such as in androgen receptor knockout (ARKO) female mice, there are only subtle impairments in adult ovarian function (Table 1). While ovarian follicle counts are similar to those in wild type mice, ARKO mice have fewer estrus cycles, smaller litter sizes, yield fewer oocytes upon gonadotropic hyperstimulation, and exhibit a diminished granulosa cell layer within follicles and smaller-sized corpora lutea [29]. Androgens may thus play important roles in the adult ovary supporting ovarian follicle and oocyte maturation, as found by Bondy and colleagues [30-32] when they treated adult female rhesus monkeys with testosterone. Unlike estrogens, however, androgens are not apparently required during fetal life for normal ovarian development.

## Fetal estrogen deficiency

Studies repeatedly implicate a key role for fetal estrogen in the normal development of ovarian morphology and function (Table 1). Exposure of fetal female baboons to a hypoestrogenic environment, by treating their pregnant mothers with an aromatase inhibitor during mid- to late gestation, halves the numbers of primordial follicles found in fetal ovaries at late gestation [33], and drastically reduces the numbers of oocyte-granulosa cell microvilli [34] that are essential for oocyte nutrition and oocyte-granulosa cell intra-follicular communication [35]. Concomitant treatment of pregnant mothers with aromatase inhibitor and estradiol prevents the late gestation ovarian consequences induced by inhibitor treatment, alone, and specifically implicates estradiol in the normal development of ovarian follicles [33,34]. Whether these fetal ovarian deficiencies translate into abnormal adult ovarian function is not yet known.

Estrogen deficiencies that are manifest beyond fetal life, as found in estrogen receptor or aromatase knockout female mice, while confounded in terms of manifesting specific fetal programming effects, provide additional insight into discrete components of estrogen action involved in ovarian development and function. ERα receptor expression is found in theca cells and ovarian stroma, while ERβ receptor is located in granulosa cells of growing follicles [36,37]. Given their differing ovarian locations, it is not surprising that the inability of estrogen to bind to the ERα receptor (ERKO) produces different abnormalities than those found in ERβ receptor knockout (BERKO) mice. ERKO female mice exhibit an ovarian phenotype of chronic anovulation, cystic and hemorrhagic follicles, absent corpora lutea, interstitial/stromal hyperplasia, and elevated plasma estradiol and testosterone levels in the presence of luteinizing hormone (LH) excess [38]. Since gonadotropin-releasing hormone (GnRH) analogue treatment normalizes LH levels and ovarian morphology in ERKO mice, and gonadotropin ovarian hyperstimulation results in ovulation, many of the ovarian abnormalities appear secondary to the loss of ERα-mediated negative feedback regulation of LH at the hypothalamus-pituitary level [39,40]. In contrast to ERKO mice, BERKO animals are ovulatory, circulating LH levels are normal, and females give birth to live young, albeit with reduced numbers of corpora lutea and smaller litter sizes [41]. The ovarian phenotype in BERKO mice thus reflects impairments in estrogen action within the ovary related to follicle maturation and differentiation [42,43]. In aromatase knockout (ArKO) mice, devoid of estrogen synthesis rather than estrogen action, the numbers of primordial follicles are reduced apparently due to their lack of formation from fetal germ cell nests and to precocious activation of follicle growth from the primordial pool [44]. In the complete absence of estrogen, ArKO females are anovulatory, folliculogenesis is arrested at the antral stage, and the ovaries manifest hemorrhagic cysts due to endogenous hypergonadotropism [45].

Unexpectedly, the adult ovarian phenotype of double estrogen receptor knockout mice (αβERKO) is masculinized, with a concomitant reduction in oocyte number. An absence of estrogen action was previously considered relatively unimportant in female mammalian differentiation [4], except for differentiation of the brain [46]. Structures resembling testicular seminferous tubules, however, are present in the ovaries of αβERKO mice, the ovaries express two genes involved in Sertoli cell differentiation, sulfated glycoprotein-2 and *Sox9*, and an additional gene involved in androgen biosynthesis, *17β-Hsd-3*, that is normally expressed only in Leydig cells [39,47]. There is even more dramatic masculinization of the ovaries of ArKO mice. Postpubertally, ArKO ovaries possess Sertoli and Leydig cells, express *Sox9*, and over-express genes coding for androgen biosynthesis [48]. Since both αβERKO and ArKO mice possess female and not male reproductive tracts, and follicles contain oocytes and not male-like germ cells, estrogen appears to play a key role in maintaining adult ovarian somatic cell phenotype [48] rather than in differentiating a fetal ovary. In humans, in comparison, while congenital aromatase deficiency leads to hyperandrogenic, multicystic ovarian phenotypes, and elevated LH and FSH levels are normalized by exogenous estrogen treatment [24], the ovaries of such women have yet to be examined for the presence of testicular structures and gene expression.

## Fetal androgen excess

In contrast to fetal androgen deficiency, fetal androgen excess has striking consequences for adult ovarian function (Table 1, [49]). Female rhesus monkeys [12], sheep [50-52], mice [53] and rats [54-56] exposed, *in utero*, to excessive levels of either testosterone or its non-aromatizable metabolite, dihydrotestosterone, exhibit intermittent or absent ovulatory cycles in adulthood. The ovaries are also enlarged and polyfollicular in prenatally androgenized monkeys and sheep [57,13], and such females have been shown to be hyperandrogenic in prenatally androgenized monkeys and mice [49,53,57]. Hypothalamic-pituitary regulation of LH release is deranged in prenatally androgen-treated females from all four species resulting in LH hypersecretion, probably driven by enhanced frequency or amplitude of hypothalamic GnRH release that, in turn, may be due to desensitization of the hypothalamus to negative feedback of estradiol and progesterone [49,53,56,58-60] and/or increased pituitary sensitivity to GnRH [49]. These androgen excess phenotypes are remarkably similar to those of women with polycystic ovary syndrome (PCOS), suggesting a fetal origin for this highly prevalent women's health disorder [61].

**Figure 1:**
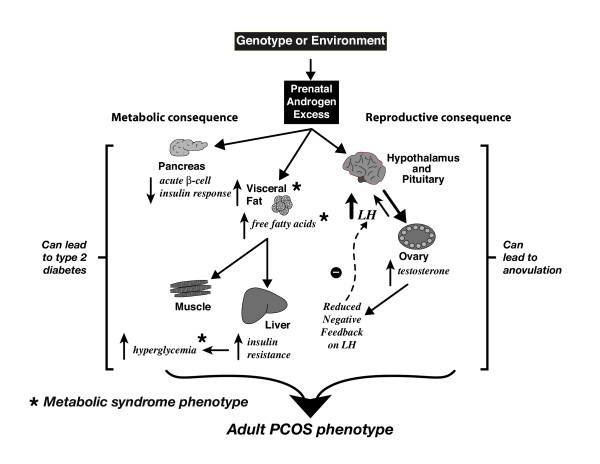
Summary of the fetal origins hypothesis for androgen excess programming of polycystic ovary syndrome (PCOS) in the prenatally androgenized female rhesus monkey, a nonhuman primate model for PCOS in women.

Administration of flutamide, an antagonist of the classical androgen receptor, to adult prenatally androgenized female mice, normalizes both hypothalamic function and cyclicity, suggestive of a role for adult hyperandrogenism in these disruptions [53]. In addition to altered androgen action, impaired insulin action may also contribute to fetal androgen excess programming of anovulation since treatment of adult, prenatally androgenized female monkeys with pioglitazone, a PPARgamma receptor ligand and insulin sensitizer, normalizes menstrual cycles [62]. In this regard, it is interesting to note that fetal androgen excess programs for metabolic dysfunction, including insulin resistance (monkeys: [49,63]; sheep: [64]) and abdominal obesity (monkeys: [65]), additional symptoms commonly found in women with PCOS, as illustrated in the fetal origins of PCOS hypothesis outlined in Figure 1. Altered glucose homeostasis and excessive insulin stimulation of ovarian follicles and oocytes may thus provide further disruption of adult ovarian function and oocyte development (see below).

In prenatally androgenized ewes, fetal testosterone excess not only leads to polycystic ovarian morphology [13], but also diminishes overall ovarian reserve and increases follicular recruitment by accelerating follicle development to primary through antral stages, thus depleting the proportion of follicles remaining in the primordial pool [66]. If such accelerated follicle recruitment continues into adult life, without compensatory reduction in follicle atresia [67] or an increase in postnatal follicle recruitment [68], premature reproductive senescence will occur, as found in three separate studies of prenatally androgenized animals [50-52]. Recent studies have found follicular persistence contributes toward the multifollicular morphology in prenatal testosterone-treated sheep [52]. Whether such ovarian abnormalities are dependent on ovarian over-stimulation by LH hypersecretion remains to be determined.

In prenatally androgenized female rhesus monkeys, controlled ovarian hyperstimulation for IVF yields oocytes deficient in developmental competence [28,69,70]. While re-initiation of meiosis, maturation and fertilization appear normal, oocytes from prenatally androgenized females progress sub-normally during the early embryonic stages. Females monkeys exposed to androgen excess during early gestation are particularly deficient and fertilized oocytes progress to blastocyst at only 13% of the numbers found in normal monkeys [28]. Intrafollicular steroidogenic responses to gonadotropic stimulation are also abnormal. Follicular fluid concentrations of androstenedione and estradiol following recombinant human (rh)FSH stimulation, alone, are diminished [69], while intrafollicular progestogenic responses to combined rhFSH stimulation followed by rhCG administration are exaggerated [28]. Since androgens [21,30,71], estrogens [72,73] and an appropriate progesterone to estradiol ratio [74,75] all enhance oocyte development in primates, the abnormal intrafollicular environment of prenatally androgenized female monkeys may contribute to their inferior oocyte quality. Dysregulation of LH and insulin secretion in prenatally androgenized females may additionally contribute to oocyte impairments [28,70].

## Fetal estrogen excess

The ovarian consequences of fetal estrogen excess are surprisingly similar to those for fetal androgen excess [76], suggesting that some components of androgen programming may be mediated by conversion of testosterone to estradiol. Since fetal female exposure to the non-aromatizable androgen, DHT, however, induces anovulation and LH hypersecretion in a similar manner to fetal testosterone excess [53,56], direct androgen action in the female fetus also re-programs adult reproductive function.

Exposure of non-primate female fetuses to diethylstilbestrol (DES), a potent estrogen, results in anovulation in adulthood [76], mainly through dysfunctional neuorendocrine regulation of hypothalamic GnRH release. There also appear to be direct effects on the ovary. In female mice exposed to DES during fetal life, adult ovarian size is diminished, follicle numbers are reduced, there is relative hyperplasia of ovarian stroma, and cultured ovarian tissue is hyperandrogenic [14,77]. Transplantation of DES-exposed fetal ovaries under the kidney capsule of ovariectomized, normal adult females fails to normalize ovarian morphology, implicating a direct ovarian effect of fetal estrogen excess programming [78]. Such fetal estrogen excess in women induces moderate hyperandrogenism [79], intermittent or absent menstrual cycles and reduced fertility [15,80,81]. Not all studies, however, find fetal DES-programmed ovarian abnormalities [82], possibly due to reduced duration of fetal DES exposure. Many of the estrogen-programmed ovarian abnormalities appear to be mediated through DES binding to ERα [83]. Such ovarian impact of *in utero *estrogen excess suggests that fetal exposure to environmental or dietary chemicals that bind to estrogen receptors may result in similar re-programming of ovarian function in adulthood [84,85]. In this latter regard, there is a potential fetal programming link between *in utero *estrogen and androgen excess: exposure of fetal rats to DES or to environmental toxicants with estrogenic activity, such as bisphenol A, increases binding activity at the androgen receptor [86]. Additionally, aromatization of androgen to estrogen may provide a crucial step in direct fetal programming of ovarian function, since fetal exposure to non-aromatizable DHT fails to induce abnormal ovarian morphology in prenatally androgenized ewe lambs [13].

## Conclusion

Sufficient fetal estrogen, but not androgen, is crucial for the normal development of oocytes and ovarian follicles. Estrogen deficiencies lead to a reduced compliment of oocytes and follicles, and abnormal ovarian function in adulthood. When estrogen deficiency is complete, such as in ArKO mice, partially masculinized ovaries develop providing a relatively new understanding of the role of estrogen in maintaining ovarian somatic cell phenotype. When estrogen deficiencies are confined to fetal life, however, it is not yet clear whether abnormalities manifest in adulthood. In contrast, discrete *in utero *excess of estrogen or androgen results in abnormal programming of ovarian function that includes anovulation and hyperandrogenism. Such reproductive abnormalities appear to involve both estrogenic and androgenic actions, since not all can be explained in terms of aromatization of androgen to estrogen. Fetal androgen excess, nevertheless, is the most clinically relevant since it closely mimics the phenotype of women with PCOS and implicates similar fetal perturbations in the developmental origin of this common human endocrine pathology.
